# Persistent inequity in maternal health care utilization in Nepal despite impressive overall gains

**DOI:** 10.1080/16549716.2017.1356083

**Published:** 2017-08-25

**Authors:** Mats Målqvist, Asha Pun, Hendrikus Raaijmakers, Ashish KC

**Affiliations:** ^a^ International Maternal and Child Health, Department of Women’s and Children’s Health, Uppsala University, Uppsala, Sweden; ^b^ UN Health Section, UNICEF, Pulchowk, Nepal

**Keywords:** Equity, maternal health care, delivery care utilization, antenatal care, Nepal

## Abstract

**Background**: Maternal health care utilization is at the core of global public health provision and an area of focus in the now-concluded Millennium Development Goal agenda.

**Objective**: This study aims to examine trends in maternal health care utilization over the last 15 years in Nepal, focusing on coverage and equity.

**Methods**: This paper used data from the Demographic Health Survey (DHS) 2001, 2006 and 2011 and Multiple Indicator Cluster Survey (MICS), 2014. Coverage rates were calculated and logistic regression models used to examine inequity.

**Results**: Impressive gains were found in antenatal care (ANC) attendance, which increased from nearly half of women attending (49%) in 2001 to 88% in 2014, and the rate of facility delivery increased from just 7–44%. This development did not, however, influence the equity gap in ANC and skilled attendance at birth, as women from low socioeconomic backgrounds were six times more likely to deliver without skilled assistance than those from high socioeconomic backgrounds (AdjOR 6.38 CI 95% 4.57–8.90) in 2014.

**Conclusion**: These persistent equity gaps call for targeted interventions focusing on the most disadvantaged and vulnerable women in order to achieve the new Sustainable Development Goal of universal health coverage.

## Background

Inequities in health have of late been increasingly highlighted and there is a growing interest in the distribution of health services and outcomes. The Sustainable Development Goals (SDGs) are an attempt to follow up on the successful creation of a united direction shaped by the Millennium Development Goals (MDGs).[1] The third SDG deals with health and places an emphasis on universal health coverage (UHC),[1] implicitly encompassing the notion of an equitable distribution of health services and outcomes. SDG 3.1 specifically mentions maternal mortality reduction. Health coverage is, however, not the same as health access, but puts a stronger emphasis on factors on the supply side than on the demand side to reach equity in health. Access to health is a more complex issue and encompasses the barriers faced by individuals and groups to reap the benefits of a health system offering universal coverage.[2] Access can be measured by utilization, assuming that all individuals would use good quality, acceptable and beneficial health services. The utilization of services thus becomes a suitable indicator of inequities within health systems and an important measure of how well the third SDG is implemented.[3]

Nepal is a low-income country that has come a long way to reduce maternal and child mortality over the last 15 years. This is despite major social challenges, with a civil war ending in 2006 and the following struggle to form a constitution and reconcile the country. The past 15 years have also been a time of economic hardship, with tourism suffering from the civil unrest as well as poor investments during the slow transition to a democratic state in this land-locked country. The under-5 mortality has declined by 44% since the year 2000, from 81/1000 to 36/1000 live births,[4] and the maternal mortality ratio (MMR) has declined from 548/100,000 live births to 258/100,000 live births over the same period.[5] Antenatal care attendance (at least one visit) in Nepal was at 85% in 2011 with a strong association with socioeconomic factors.[6] Only half of women attended the recommended number of four or more visits.[6] Dedicated efforts to increase access to care in the most isolated and hard-to-reach areas of this mountainous country have, however, been put in place, with a decrease in home delivery rates as a result.[7] An ambitious program deploying Female Community Health Workers in order to increase coverage of maternal and child health care interventions has been claimed to be part of this success.[8] There are, however, many challenges remaining. The neonatal mortality rate has declined at a slower pace, 34% over the same period, and now constitutes an increasing proportion of the overall child mortality. There are also indications that the improvements in maternal health care utilization have not been equitable and that inequities still persist.[9] The objective of this study is therefore to investigate the development of maternal health care utilization as defined by antenatal care coverage, skilled attendance at birth and postnatal care, and to monitor changes in equity for these indicators.

## Methods

Data from DHS 2001, 2006 and 2011 and MICS5 from 2014 were sourced for the study. Women who had given birth prior to the surveys were included, those within five years prior in DHS and two years prior in MICS5. Utilization of health services in connection to the latest pregnancy was investigated. Both DHS and MICS5 used multi-stage stratified sampling procedures; interviewing women to collect data on their reproductive health history. We limited our analysis to women between 15 and 49 years of age who had delivered a live birth within the period of study. Data from the most recent delivery were used. The sample size for the DHS data was 4745, 4066 and 4079, respectively, and the sample size for the MICS5 survey was 2086 (Table 1). Sampling procedures have been described elsewhere and can be considered representative for Nepal at large.[10–13] DHS data were accessed with the permission of ORC/MACRO, and MICS5 data were accessed with permission from UNICEF.

**Table 1.** Characteristics of women who had given birth within two (MICS) and five (DHS) years preceding survey in Nepal.

	DHS 2001	DHS 2006	DHS 2011	MICS 2014
***N***	4745	4066	4079	2086
**Personal characteristics**				
Age (mean)	27.73	26.98	26.94	25.72
	*n* (%)	*n* (%)	*n* (%)	*n* (%)
Primipara	993 (20.9)	1094 (26.9)	1248 (30.6)	784 (37.6)
Multipara	3753 (79.1)	2972 (73.1)	2831 (69.4)	1302 (62.4)
Married	4695 (98.9)	4006 (98.5)	4033 (98.9)	2064 (99.5)
Single	50 (1.1)	60 (1.5)	46 (1.1)	22 (0.5)
**Socioeconomic characteristics**				
*Wealth quintiles*				
5th Wealthiest	710 (15.0)	687 (23.5)	671 (16.5)	254 (12.2)
4th	923 (19.4)	752 (18.5)	677 (16.6)	443 (21.2)
3rd	921 (19.4)	811 (20.0)	739 (18.1)	311 (14.9)
2nd	1012 (21.3)	859 (21.1)	832 (20.4)	443 (21.2)
1st Poorest	1180 (24.9)	956 (23.5)	1160 (28.4)	762 (36.5)
*Education*				
Literate	1628 (34.3)	2044 (50.3)	2595 (63.6)	1264 (60.6)
Illiterate/Never attended school	3117 (65.7)	2022 (49.7)	1484 (36.4)	822 (39.4)
*Living area*				
Hill/Terai	4385 (92.4)	3726 (91.6)	3337 (81.8)	1470 (70.5)
Mountain	361 (7.6)	340 (8.4)	742 (18.2)	616 (29.5)
Urban	332 (7.0)	536 (13.2)	897 (22.0)	343 (16.4)
Rural	4414 (93.0)	3530 (86.8)	3182 (78.0)	1743 (83.6)

### Main outcome variables

Three main areas in the continuum of care were investigated; antenatal care coverage, skilled assistance at delivery, and postnatal care. For antenatal care coverage, it was investigated whether the included women had attended ANC at least once, in the first trimester, four times or more, and if the ANC visit (any) had been attended by a skilled provider (doctor, midwife/nurse). For delivery care utilization, we included place of delivery and whether delivery had been attended by a skilled provider (doctor, midwife/nurse). Postnatal care was measured both through selected quality indicators; whether breastfeeding was initiated within the first hour after delivery or not, as well as through coverage measures indicating a stay at the health facility and whether the mother and baby received health checkups both after delivery and within the first week after delivery (day 1–6).

### Indicators of inequity

In order to analyze equity gaps in relation to these main outcome variables the theoretical framework set up by the Commission on Social Determinants of Health (CSDH) guided the analysis and choice of variables.[14] The CSDH framework emphasizes social position, as decided by a number of structural determinants, as the main driver of inequities in health. We thus included wealth status and education level of mother measured by literacy in the analysis. The ethnicity of the household head and religious beliefs were not available in MICS5 and we thus excluded these structural variables for reasons of comparison. We deviated from the CSDH framework by including place of residence as a structural determinant. In the CSDH framework, living area is considered a proximal determinant, but, given the inaccessible geography of Nepal’s mountainous regions, as well as the results of recent research that focuses on the urban-rural divide, we also incorporated these variables as potential drivers of inequity.

Wealth, or socioeconomic status, was defined through a pre-set wealth index based on assets and was calculated by principal components analysis as defined in the respective survey methodologies.[15] The mother’s education level was defined as either the mother being illiterate or literate.

### Data analysis

Coverage of the selected variables for maternal health care utilization was calculated and divided by the structural determinants described above. All analyses were adjusted for sample survey weights. The chi-squared test was used to detect group differences and a *p*-value of <0.05 was considered significant. A multivariable logistic regression analysis was then applied with all independent variables included. Adjusted odds ratios and 95% confidence intervals were calculated. All analyses were performed in SPSS 20.0.

## Results

Over the years there was a slight decrease in the mean age of mothers interviewed and an increase in the proportion of women who had not previously given birth (primipara women). Basically, most women were married in all four surveys, and thus this variable was excluded from further analyses. When looking at the socioeconomic variables, is it worth noting that the proportion of illiterate women decreased dramatically; from 65.7% in 2001 to 39.4% in 2014 (*p *< 0.001). A contrary trend could be seen in the proportions of poor mothers giving birth, which rose from 24.9% in 2001 to 36.5% in 2014 (*p *< 0.001), suggesting a declining fertility rate among those mothers of higher socioeconomic status (Table 1).

Impressive gains in maternal health care can be seen in the material, with antenatal care attendance increasing from just about half of the women attending (49.1%) in 2001 to 88% in 2014, and the rate of facility delivery increasing from only 7% to almost half (44%) of deliveries taking place at a health facility (Table 2). The largest gains in antenatal care can be seen in the time period up until 2011, after which it stabilizes. Not only did the rate of antenatal care attendance increase, but also the proportion of women coming to ANC in the first trimester as well as the proportion of women attending four or more visits, which increased considerably.

**Table 2.** Maternal health care utilization over time in Nepal. Data from DHS 2001, 2006, 2011 and MICS5 2014. Coverage adjusted for clustering.

	2001		2006		2011		2014	
**Antenatal care**	*N* = 4745	Weighted % (95% CI)	*N* = 4066	Weighted % (95% CI)	*N* = 4079	Weighted % (95% CI)	*N* = 2086	Weighted % (95% CI)
Attended ANC	2332	49.1 (47.6–50.7)	3000	73.8 (72.2–75.3)	3468	84.8 (83.5–86.1)	1766	87.8 (85.9–89.4)
ANC in first trimester	780	16.4 (15.3–17.6)	1127	37.6 (35.4–39.8)	2061	58.5 (56.5–60.5)	920	50.8 (48.1–53.5)
ANC 4 times or more	689	14.5 (13.5–15.6)	1198	29.5 (27.7–31.3)	2151	50.1 (48.2–52.0)	1196	59.5 (56.8–62.1)
ANC with skilled provider (doctor, midwife/nurse)	1325	27.9 (26.6–29.3)	1777	43.7 (41.9–45.6)	2502	58.3 (56.4–60.1)	1312	68.3 (65.8–70.7)
At least 2 tetanus vaccinations before delivery	2158	45.5 (43.3–47.0)	3371	83.3 (82.0–84.5)	2805	69.9 (68.2–71.6)	1567	78.3 (76.1–80.4)
**Delivery**								
Home delivery	4194	88.4 (87.4–89.3)	3221	79.2 (77.8–80.7)	2397	59.8 (57.9–61.6)	969	43.1 (40.4–45.8)
Hospital delivery (government or private)	330	7.0 (6.3–7.8)	695	17.1 (15.7–18.6)	1231	29.4 (27.7–31.1)	774	44.4 (41.7–47.2)
Delivery at private provider	54	1.1 (0.9–1.5)	161	4.0 (3.3–4.8)	388	10.1 (9.0–11.3)	179	10.6 (9.0–12.5)
Attended by skilled provider (doctor, midwife/nurse)	553	11.6 (10.7–12.6)	863	20.6 (19.1–22.2)	1950	39.1 (37.3–41.0)	844	48.8 (46.1–51.5)
**Postnatal care**								
Early initiation of breastfeeding (immediately or within 1 hour)	1463	31.2 (29.9–32.7)	1423	35.4 (33.6–37.2)	1632	44.7 (42.8–46.5)	1073	50.3 (47.6–53.1)
Staying more than 24 hours at health facility	N/A*		651	67.5 (63.3–71.5)	N/A*		744	73.5 (70.3–76.5)
Baby’s health checked after delivery by trained health professional	666	14.0 (12.9–15.2)	1098	27.9 (26.2–29.6)	1578	38.4 (36.6–40.3)	810	47.9 (45.2–50.6)
Baby’s health checked at least once in first week (day 1–6) by trained health professional (before leaving hospital or at home)	N/A°		727	16.6 (15.2–18.1)	N/A°		427	24.4 (22.1–26.8)

*Variable not included in dataset

°Insufficient data

Also, the postnatal care displayed some overall improvements. The rate of mothers initiating breastfeeding within one hour after delivery rose from 31% in 2001 to 50% in 2014. The number of babies being checked by a health professional after delivery was still low in 2014, with less than half (48%) of the newborns being examined (Table 2). This is, however, a higher rate than in 2001, when only 14% were checked, a development that, to a large extent, can be explained by the increased facility delivery rate.

For further analyses, ANC attendance and skilled attendance at birth (SBA) was used. The rate of SBA followed the trend of facility delivery, but it can be noted that the percentage of deliveries with SBA did not match the facility delivery rate in 2014, implying that SBA was not available at all facility deliveries.

**Figure 1.**
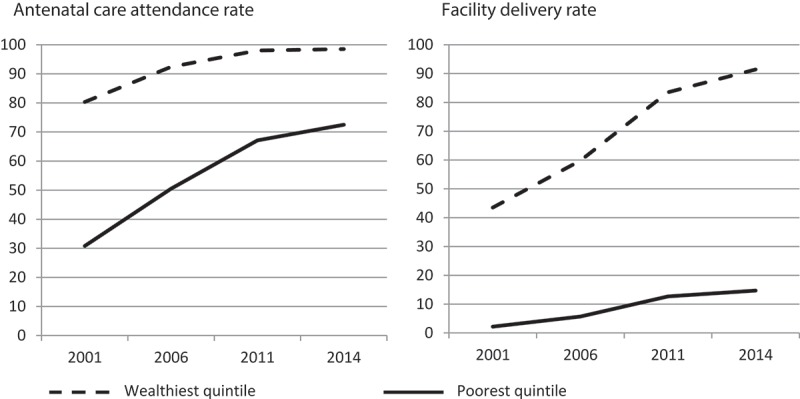
Equity gap in antenatal care attendance and facility delivery rate 2001–2014 in Nepal.

Despite these overall gains in maternal health care utilization in Nepal, the equity gaps persist. Figure 1 illustrates how the upward trend in ANC attendance and facility delivery rate has occurred with a maintained gap in utilization between the poorest and the wealthiest populations. Logistic regression analyses relating the dependent variables ANC attendance and SBA to socioeconomic factors revealed a sharp inequity based on wealth, education and living area. All socioeconomic variables showed a significant association with the dependents in a univariate analysis and were included in two multivariate logistic regression models (Table 3). The first regression model, which investigated associations with ANC attendance, revealed a persistent inequity, with all confidence intervals overlapping over all four time points except for the rural/urban divide, where an improvement was seen between 2001 and 2006. Overall, the risk for mothers from families of low socioeconomic status and who were illiterate to not attend ANC during pregnancy was around three-fold compared to their peers from families of higher socioeconomic status (Table 3). The second regression model with SBA also showed a maintained inequity gaps for all of the examined structural determinants (confidence intervals overlapping). The largest difference was detected for the urban–rural divide, with a woman living in rural Nepal in 2001 being almost seven times more likely to not deliver with SBA compared to her peer in an urban area (adjOR 6.82, CI95% 5.34–8.70). This risk was reduced in 2006 and 2011, but rose again in results from the MICS5 in 2014 to an even higher level than in 2001 (Table 3).

**Table 3.** Multivariate logistic regression displaying adjusted odds ratios for maternal health care utilization (women not receiving ANC during latest pregnancy and delivering without skilled birth attendance) in Nepal, adjusted for maternal age.

	2001		2006		2011		2014	
***No ANC attendance***								
	OR	CI 95%	OR	CI 95%	OR	CI 95%	OR	CI 95%
Non-poor (2nd–5th percentile)	Ref		Ref		Ref		Ref	
Poor (lowest percentile)	2.17	1.87–2.52	2.93	2.48–3.46	2.82	2.31–3.43	2.78	2.09–3.73
Literate	Ref		Ref		Ref		Ref	
Illiterate	3.02	2.63–3.46	2.21	1.88–2.60	2.38	1.95–2.92	2.16	1.62–2.89
Hill/Terai	Ref		Ref		Ref		Ref	
Mountain	1.70	1.33–2.19	1.44	1.12–1.85	1.16	0.93–1.46	1.24	0.93–1.65
Urban	Ref		Ref		Ref		Ref	
Rural	3.15	2.63–4.27	1.68	1.27–2.23	1.61	1.20–2.16	1.98	1.17–3.36
***No skilled attendance at birth***								
	OR	CI 95%	OR	CI 95%	OR	CI 95%	OR	CI 95%
Non-poor (2nd–5th Percentile)	Ref		Ref		Ref		Ref	
Poor (lowest percentile)	3.62	2.66–4.94	3.31	2.54–4.32	3.60	3.04–4.25	5.42	4.17–7.04
Literate	Ref		Ref		Ref		Ref	
Illiterate	4.48	3.74–5.35	3.84	3.24–4.54	2.63	2.29–3.05	2.52	1.98–3.12
Hill/Terai	Ref		Ref		Ref		Ref	
Mountain	1.73	1.09–2.72	1.70	1.17–2.47	1.80	1.50–2.16	2.13	1.64–2.77
Urban	Ref		Ref		Ref		Ref	
Rural	6.58	5.27–8.19	4.13	3.44–4.95	3.54	3.03–4.14	7.46	5.29–10.5

## Discussion

This study shows how gains in overall maternal and child health utilization in Nepal have not reduced the equity gap in relation to the household’s socioeconomic status and maternal education level. These findings are in line with previous investigations of the distribution of health care utilization over time,[16,17] where improvements happen simultaneously in various groups in society.[18] The tendency for the health gains to be realized earlier in urban areas and among those of high socioeconomic status indicates a need for targeted interventions to reach the most disadvantaged and vulnerable.[19,20] As with the successful immunization efforts to reach the hard-to-reach and increase immunization rates through reducing inequity,[21] similar strategies to identify neglected geographical areas for intensified efforts could also be applied to maternal health care utilization.

This study performed a secondary analysis of pre-existing data and the data used are considered representative for Nepal. There are, however, some limitations to the analyses based on differences between the different surveys. MICS5 has, for example, no data on ethnicity or caste, structural determinants that would have been interesting to add to the analysis. Ethnicity and caste could potentially be confounding factors, but the overall message from the result would most likely not be changed. Measuring wealth is a complex task and one commonly used method is to create asset indices, with or without a principal component analysis. This method has some limitations, especially when comparing urban and rural areas, as assets vary with access. It does, however, provide a crude estimation of the socioeconomic status of respondents and is sufficient for the analysis at hand. Another limitation of this study is the difference between questionnaires and the phrasing of questions. The set of variables differ somewhat in all surveys, and, when conducting a comparison, this poses a limit to what can be examined. Furthermore, the sampling frames for DHS and MICS are not identical, which can compromise comparability. The recall period is also longer in the DHS material (five years) compared to in the MICS (two years), which could result in different levels of recall bias.

A secular trend, i.e. an overall development in society, is visible in the material, with increased literacy rate and a shift from rural to urban dwelling for an increasing part of the population. The increase in overall education level can explain the trend of the closing (however not significantly) equity gap based on maternal literacy. The increasing rate of care utilization over time can also partly be attributed to a larger proportion of the population living in urban areas with shorter distances to health facilities. It is, however, notable that the home delivery rate is still high in Nepal despite this secular trend and the increased efforts made as part of the steps towards MDGs to encourage pregnant women to come to health facilities to deliver. Current results show that there is still plenty of room for improvement to understand the bottlenecks of the system. Issues such as why women who attend antenatal care do not follow through and deliver at a facility or with a skilled provider need to be investigated, and more emphasis on facility delivery and its advantages need to be placed early during ANC. Experiences of care previously received, including being subject to disrespect and abuse, influence the decision to seek care [22] and therefore further studies investigating mothers’ perceptions of care received are needed.

Skilled assistance at birth has previously been used as a proxy for maternal mortality and is one of the basic services provided by all health systems.[23] The continuum of care during pregnancy and childbirth is an essential part of every health system and is crucial in the struggle to fight maternal and child mortality,[24,25] even if recently it has been acknowledged that increasing access and availability is not enough.[26] There is also a need to focus on quality of care (QoC).[23,27] It can fairly be assumed that there is a further aggravation of the equity gap if also including QoC, with the better off not only accessing care more frequently, but also receiving better care when doing so. WHO has recommended *Standards for improving quality of maternal and newborn care in health facilities* [28] in an effort to highlight the need to secure a good QoC for all. How these standards are going to be implemented is still a challenge that has received increasing attention. Although there have been multiple studies on quality improvement interventions, there is a dearth of evidence on how to scale up these efforts within health systems, something that is necessary if the equity gap is to be addressed.

The role of antenatal care (ANC) to promote healthy lives in a life course perspective and the importance of skilled attendance at birth to reduce child mortality are at the core of health service provision in low- and middle-income countries.[3] Similarly, the high burden of home deliveries and unskilled attendance at birth is a major challenge for postnatal care, not only in regards to accessibility, but also in relation to quality of care.[29] It has previously been shown how inequities in maternal health care utilization not only depend on socioeconomic status but also on maternal education and ethnicity.[30] Its strong association with maternal and child mortality makes maternal health care utilization a prioritized area for monitoring when it comes to universal health coverage.[24,25]

## Conclusion

To compare results from population-based surveys over time can yield important insights about the success or failure of health policy and planning. Through a rather simple and straightforward equity analysis, health systems can be informed about the need for specific action beyond aggregated effectiveness measures. As an example, our results show how impressive gains have been made in maternal health care utilization in Nepal, but also how these improvements have happened while maintaining equity gaps. This indicates that there is still a need for targeted interventions as part of achieving universal health coverage.

## Paper context

This study analyzes data from nationwide surveys in Nepal from 2001 to 2014. Results show how impressive gains have been made in maternal health care utilization in Nepal, but also how these improvements have happened while maintaining equity gaps. This demonstrates how there is a need to focus on disadvantaged and vulnerable groups to reduce inequity and achieve universal health coverage as part of the Sustainable Development Goal agenda.
